# TIPS: a system for automated image-based phenotyping of maize tassels

**DOI:** 10.1186/s13007-017-0172-8

**Published:** 2017-03-31

**Authors:** Joseph L. Gage, Nathan D. Miller, Edgar P. Spalding, Shawn M. Kaeppler, Natalia de Leon

**Affiliations:** 1 Department of Agronomy, University of Wisconsin-Madison, 1575 Linden Drive, Madison, WI 53706 USA; 2 Department of Botany, University of Wisconsin-Madison, 430 Lincoln Drive, Madison, WI 53706 USA

**Keywords:** *Zea mays* L., Tassel, Image analysis, Phenotyping, High-throughput, Inflorescence

## Abstract

**Background:**

The maize male inflorescence (tassel) produces pollen necessary for reproduction and commercial grain production of maize. The size of the tassel has been linked to factors affecting grain yield, so understanding the genetic control of tassel architecture is an important goal. Tassels are fragile and deform easily after removal from the plant, necessitating rapid measurement of any shape characteristics that cannot be retained during storage. Some morphological characteristics of tassels such as curvature and compactness are difficult to quantify using traditional methods, but can be quantified by image-based phenotyping tools. These constraints necessitate the development of an efficient method for capturing natural-state tassel morphology and complementary automated analytical methods that can quickly and reproducibly quantify traits of interest such as height, spread, and branch number.

**Results:**

This paper presents the Tassel Image-based Phenotyping System (TIPS), which provides a platform for imaging tassels in the field immediately following removal from the plant. TIPS consists of custom methods that can quantify morphological traits from profile images of freshly harvested tassels acquired with a standard digital camera in a field-deployable light shelter. Correlations between manually measured traits (tassel weight, tassel length, spike length, and branch number) and image-based measurements ranged from 0.66 to 0.89. Additional tassel characteristics quantified by image analysis included some that cannot be quantified manually, such as curvature, compactness, fractal dimension, skeleton length, and perimeter. TIPS was used to measure tassel phenotypes of 3530 individual tassels from 749 diverse inbred lines that represent the diversity of tassel morphology found in modern breeding and academic research programs. Repeatability ranged from 0.85 to 0.92 for manually measured phenotypes, from 0.77 to 0.83 for the same traits measured by image-based methods, and from 0.49 to 0.81 for traits that can only be measured by image analysis.

**Conclusions:**

TIPS allows morphological features of maize tassels to be quantified automatically, with minimal disturbance, at a scale that supports population-level studies. TIPS is expected to accelerate the discovery of associations between genetic loci and tassel morphology characteristics, and can be applied to maize breeding programs to increase productivity with lower resource commitment.

**Electronic supplementary material:**

The online version of this article (doi:10.1186/s13007-017-0172-8) contains supplementary material, which is available to authorized users.

## Background

The male inflorescence (tassel) of maize (*Zea mays* L.) is a branched structure atop the plant that produces pollen and complements the female inflorescence (ear) to enable reproduction. Tassel size and morphology have implications for the amount of pollen produced, which can affect maintenance of inbred lines, hybrid production, and subsequent agricultural yields. Though a certain level of pollen production is necessary for these reasons, decreased tassel size has been correlated with an increase in grain yield over the last half century of modern maize breeding [1]. Large tassels have been shown to decrease light interception by the upper leaves of maize plants and have been correlated with decreases in grain yield, effects that are exacerbated by higher planting densities [2, 3]. Genomic regions controlling tassel morphology have been described in the literature, but those studies have traditionally focused on simple, quantifiable, hand-measured traits such as tassel length, number of branches, and length of the zone where branches originate [4–7]. This focus on traits that are easy and quick to measure is due to the fact that tassels are large and relatively delicate. While ears can be dried and still maintain their overall shape, making it possible to store and analyze them at the convenience of the researcher, tassels are fragile and easily deformed within hours of removal from the living maize plant. As a result, in order to study many of the shape characteristics of tassels they must be measured directly in the field.

Though the studies listed above have investigated tassel morphology, the questions of how tassel shape and size affect pollen production and grain yield have yet to be answered conclusively. Related genes controlling phenotypic variation have also not been fully characterized. Additionally, all studies to date have dealt with traits that can be measured by hand on dried tassels, so there is opportunity to explore tassels more comprehensively using new technologies.

Increasing availability of computational resources has enabled large scale studies utilizing image analysis, and there has been growing interest in high-throughput phenotyping in crops in general. Therefore, we chose to study tassel architecture by image analysis. A few solutions already exist that can be utilized to process images of maize tassels. PANorama and P-Trap [8, 9] were developed for image analysis of rice panicles using images obtained by flatbed scanner or by arranging the panicle on a flat board. Furthermore, PANorama can process images of organs from other plant species, including maize tassels. Software built for measuring similar features (branching structure, lengths, curvature, etc.) have been developed for analyzing images of plant roots. Tassels, when inverted, bear morphological similarity to root systems therefore software such as GiA Roots and DIRT [10, 11] might represent an alternative for the analysis of tassels. This study compared results from GiA Roots, DIRT, and PANorama to our novel Tassel Image-based Phenotyping System (TIPS) for the analysis of maize tassels.

The main objectives behind creating TIPS were to develop a platform for rapidly imaging large numbers of tassels in a field setting, to develop image analysis methods to corroborate hand-measured traits, and to demonstrate examples of image-based phenotyping tools to quantify traits that otherwise would be difficult, impossible, or time-consuming to measure by hand.

## Methods

### Genetic material, plant growth conditions, and sample collection

A set of 749 inbred lines from an expanded version of the Wisconsin Diverse Panel (WiDiv) [12] were grown at the West Madison Agricultural Research Station in the summer of 2015. Lines were planted in a randomized complete block design with two field replications. The experiment was planted in 4.57 m long single row plots with 0.76 meters between rows at a density of 72,000 plants per hectare. Tassels were collected from three representative plants per plot by cutting them 10.8 cm below the lowest tassel branch. Tassels were collected from a plot for imaging when half of the plants in the plot had extruded anthers, but whenever possible the sampled tassels were taken from plants that had not yet themselves extruded any anthers. This ensured that the collected tassels were as developmentally close to flowering as possible, but avoided having exposed anthers in the images. Samples were carried to the margin of the field, where they were subsequently imaged.

### Sample imaging

Tassels were imaged using portable photography boxes made from PVC frames with white floors and backgrounds. Interior dimensions of the photography boxes were 91.5 cm wide, 122 cm tall and 61 cm deep. Tassels were mounted upright in the center of the photography box floor. Images were captured using a Nikon D3300 DSLR camera with an 18–55 mm lens set to 18 mm. The camera was mounted to a fixed boom that was part of the PVC framework, ensuring a consistent angle and distance (76 cm) relative to the samples. The camera was attached to a laptop computer and controlled by custom gphoto2 scripts that captured images and wrote them directly to the hard drive. For each sample, a background image was taken of the empty photography box, followed by an image with the sample present, resulting in two images per sample. Tassels were always oriented such that any curvature along the main spike was in the plane perpendicular to the camera’s optical axis. Image dimensions were 4000 × 6000 pixels.

### Manual measurements

**Fig. 1:**
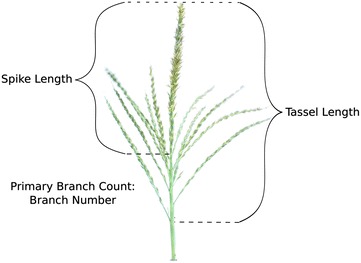
Manually measured traits. An example of manually measured tassel traits. Tassel length is the distance from the lowermost branch to the spike tip. Spike length is the distance from the uppermost branch to the spike tip. Branch number is a count of primary branches, i.e., those that intersect the central axis of the tassel, and not those that originate on another branch. Not pictured is tassel weight, which was measured as the average weight of three dried tassels per plot

Immediately following image acquisition, tassels were measured manually for four traits: tassel length, spike length, branch number, and tassel weight (Fig. 1). Tassel length was measured as the distance (mm) from the lowest branch point to the tip of the tassel. Spike length was measured as the distance (mm) from the highest branch point to the tip of the tassel. Branch number is a count of the number of primary tassel branches. All three sample replicates from a single plot were dried and weighed together to obtain a plot mean tassel weight in grams.

### Repeatability, correlations, and coefficient of variation of root mean squared error

Repeatability was calculated for each trait by fitting a linear model of y = u + G + B + e, where y represents the trait values for individual plants, u represents an overall mean, G represents a genotype effect, B represents a field replication effect, and e represents residual effects. G was modeled as random, while B was fixed. Repeatability was calculated as σ_G_^2^/(σ_G_^2^ + [σ_e_^2^/b]), where σ_G_^2^ is the genotype variance, σ_e_^2^ is the residual variance and b is the number of field replications (two in this case).

Pearson correlations $\left( {r = \frac{{cov\left( {x, y} \right)}}{{\sigma_{x} \sigma_{y} }}} \right)$ between traits were calculated using genotype means for each trait across replications. Coefficient of variation of root mean squared error [CV(RMSE)] was calculated for each hand measured trait as the square root of the mean squared error of the regression of hand-measured values on image-based values, divided by the mean of the hand-measured values. Image-based measurements of tassel length were converted from pixels to millimeters using the base of the tassel holder for scale.

### Pre-existing image analysis software

An arbitrary sample of 200 images, each representing a different genotype, were selected to be tested with two popular root-analysis programs (GiA Roots and DIRT) [10, 11] and an existing inflorescence analysis program (PANorama) [8] to evaluate the need for a specially written tassel analysis program. For DIRT and GiA Roots, images were flipped vertically and the grayscale color profile inverted to resemble light roots on a dark background. Both original RGB images as well as RGB images with the background removed were used as input for PANorama. The output from DIRT, GiA Roots and TIPS were tested for correlations to hand-measured phenotypes to determine the need for tassel-specific image analysis methods. Differences between the correlations were tested with a two-sided test for equivalence of dependent correlations as implemented in the R package ‘psych’ [13].

### Computational methods and tools

TIPS was written in the MATLAB programming language and returns fully automated image-based measurements of tassel length, branch number, tassel area, tortuosity, compactness, fractal dimension, skeleton length, and perimeter length. It also returns a binary image of each tassel with some of the above traits illustrated in color, which can be used for quality control or illustration. All downstream analysis of results was performed in R [14].

Scripts for image and analysis can be found on Github at http://github.com/joegage/TIPS.git and a set of 200 sample images can be found at http://phytomorph.wisc.edu/download/TIPS.

## Results

**Fig. 2:**
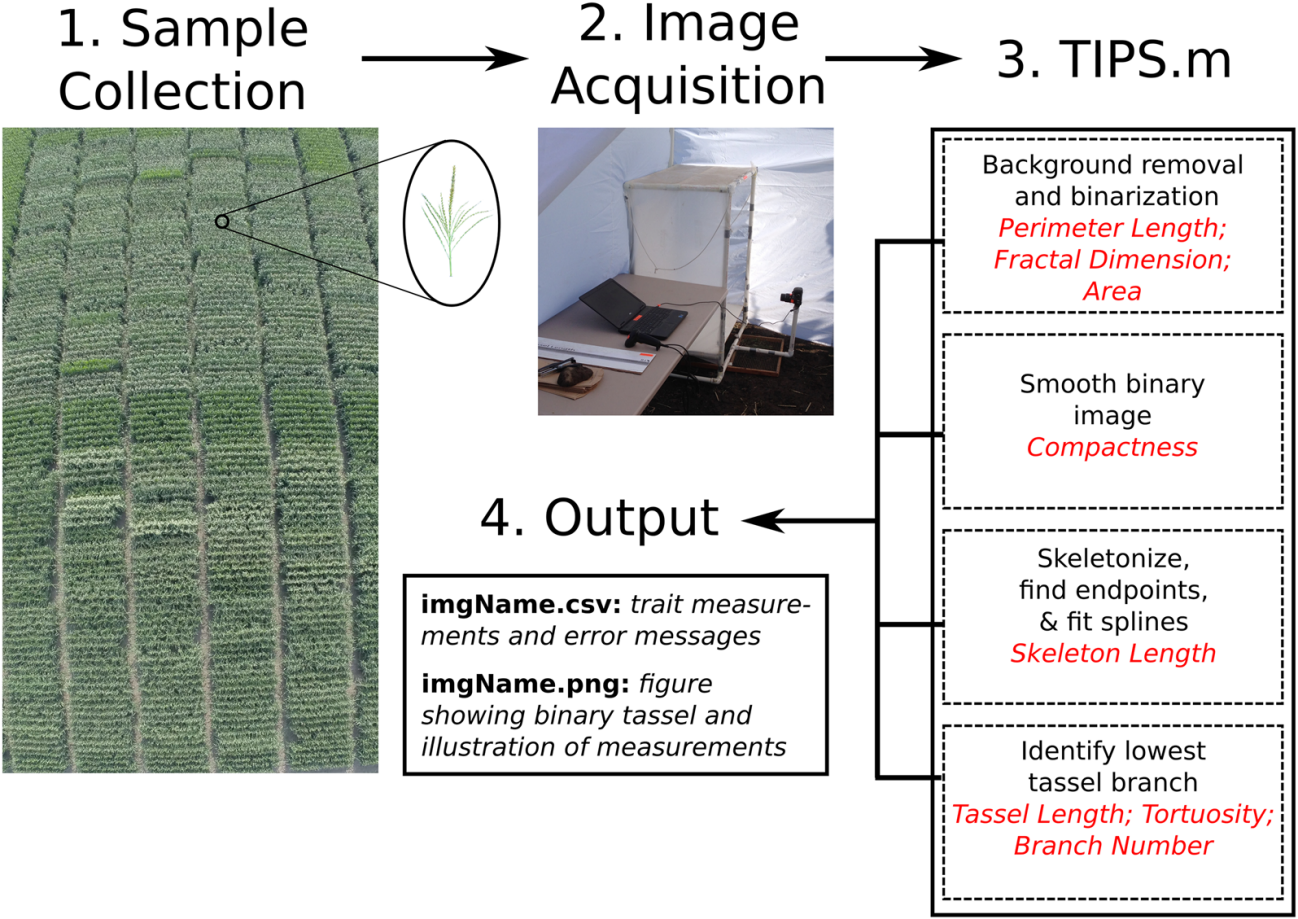
TIPS Flowchart. Tassels were collected from replicated field plots (*1*) and imaged in a PVC photography booth (*2*) using automated gphoto2 scripts. Image analysis by TIPS software (*3*) removed background noise, then binarized, smoothed, and skeletonized the tassel. Each analysis step is listed in *black type*, while the trait measurements resulting from that step are displayed in *red type*. The trait measurements were returned in a .csv file along with a figure visually showing some of the measurements (*4*)

A collection of 3530 maize tassels representing 749 diverse inbred lines was imaged and processed using TIPS. Tassels were manually removed from plants, photographed immediately, and the images were subsequently analyzed by TIPS. In short, the process of image analysis involved removing background noise and binarizing the image; smoothing, skeletonizing, and fitting splines to the binary image; and identifying the start point of the lowest tassel branch. Figure 2 presents a flowchart showing these steps and the output from each. Since a major goal of this project was to maximize throughput, a single 2D image was taken of each tassel.

Substantial variability was observed for all hand and image based measurements (Table 1). TIPS can measure branch number, tassel length, and tassel weight with moderate or high correlations to hand measurements, and has high enough throughput to image tassels at a rate of approximately 20 tassels per person per hour, including collection from the field, image acquisition, and hand measurement of tassel length, spike length, and branch number. A number of parameters can be passed in vector form to TIPS. Default values were chosen heuristically for these analyses but are accepted as optional arguments by TIPS to enable parameter sweeps for individual populations or images (Additional file 1: Table S1).

**Table 1:** Minimum, maximum, and repeatability of all measurements

Trait	Minimum	Maximum	Repeatability
Weight^a^ (g)	3.31	51.13	0.85
Tassel length^a^ (mm)	211.17	538.33	0.88
Spike length^a^ (mm)	111.5	369.33	0.89
Branch number^a^	0	40.83	0.92
Area^b^ (mm^2^)	2225.01	16,507.89	0.83
Branch number^b^	1.50	17.33	0.77
Tassel length^b^ (mm)	166.28	469.48	0.77
Tortuosity^b^	0.75	1.00	0.49
Compactness^b^	0.07	0.76	0.77
Fractal dimension^b^	1.26	1.53	0.81
Skeleton length^b^ (mm)	260.38	2261.98	0.78
Perimeter length^b^ (mm)	567.72	3034.39	0.71

Range of phenotypic values and repeatability for all manual and image-based trait measurements. Traits noted by ^a^ were measured by hand, traits noted by ^b^ were measured by image-based method. Minimums and maximums represent genotype means across two replications

### Pre-processing of tassel images

**Fig. 3:**
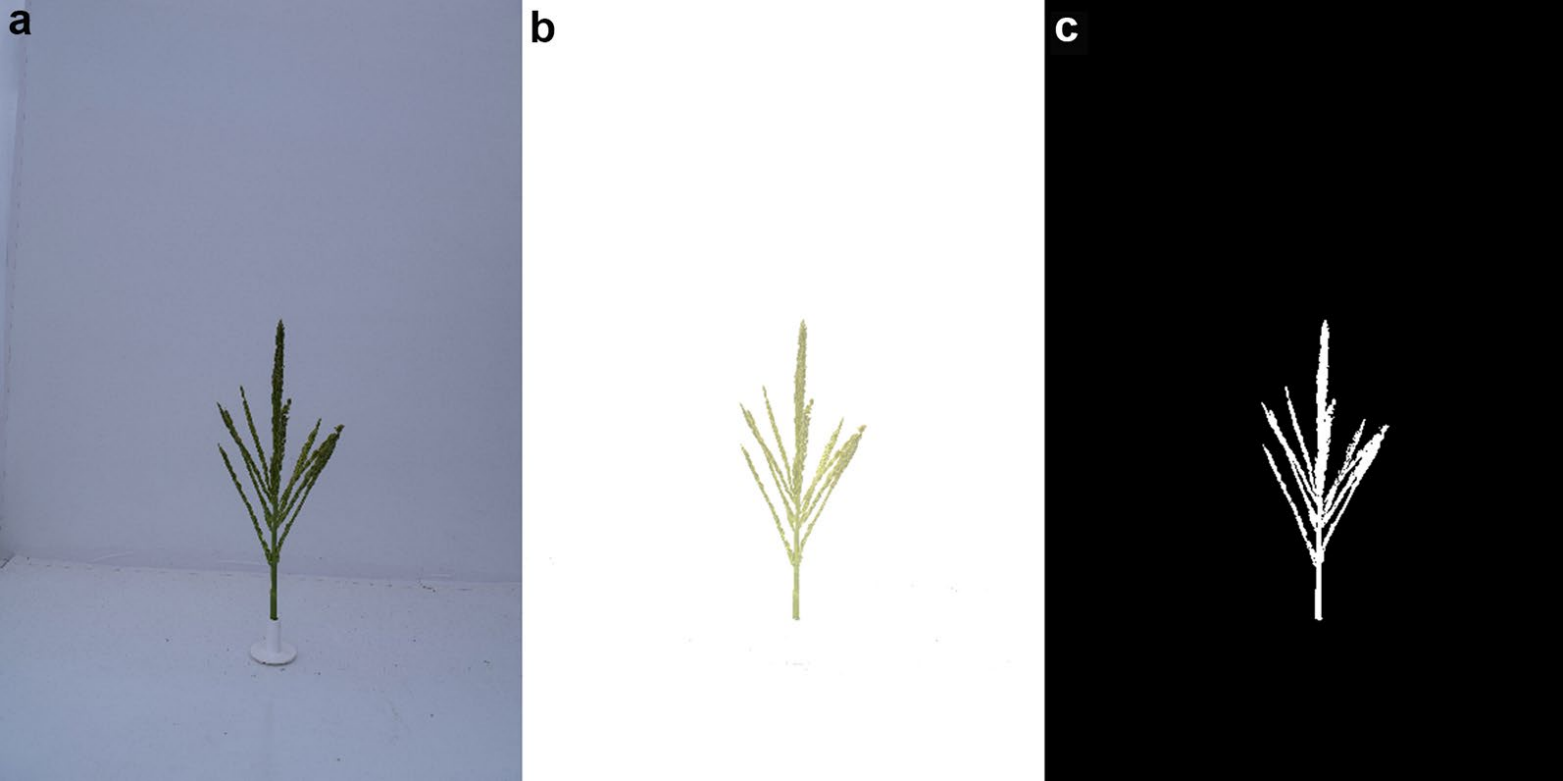
Image background removal and binarization. The original image (**a**) has the background subtracted and any remaining artifacts removed, resulting in (**b**), which is then thresholded to produce the binary image in (**c**)

Immediately before each tassel was imaged, a background image was taken to capture ambient light and background debris in the photo booth. Figure 3 shows the effects of subtracting the background image from the image containing the tassel, then converting it to a binary form by a standard threshold method [15]. Only the largest continuous object in the binary image was retained. This filtering step removed any small artifacts not removed by background subtraction (e.g., debris, reflections, dirt). Images were excluded from further analysis if the automatically chosen threshold value was lower than an empirically determined minimum, which would be the case if there was no tassel in the image, or if poor contrast in the original image prevented a faithful threshold operation. Images were also removed from further analysis if any portion of the final binary image contacted the border (indicating the tassel extends outside the frame of the image).

### Tassel length

**Fig. 4:**
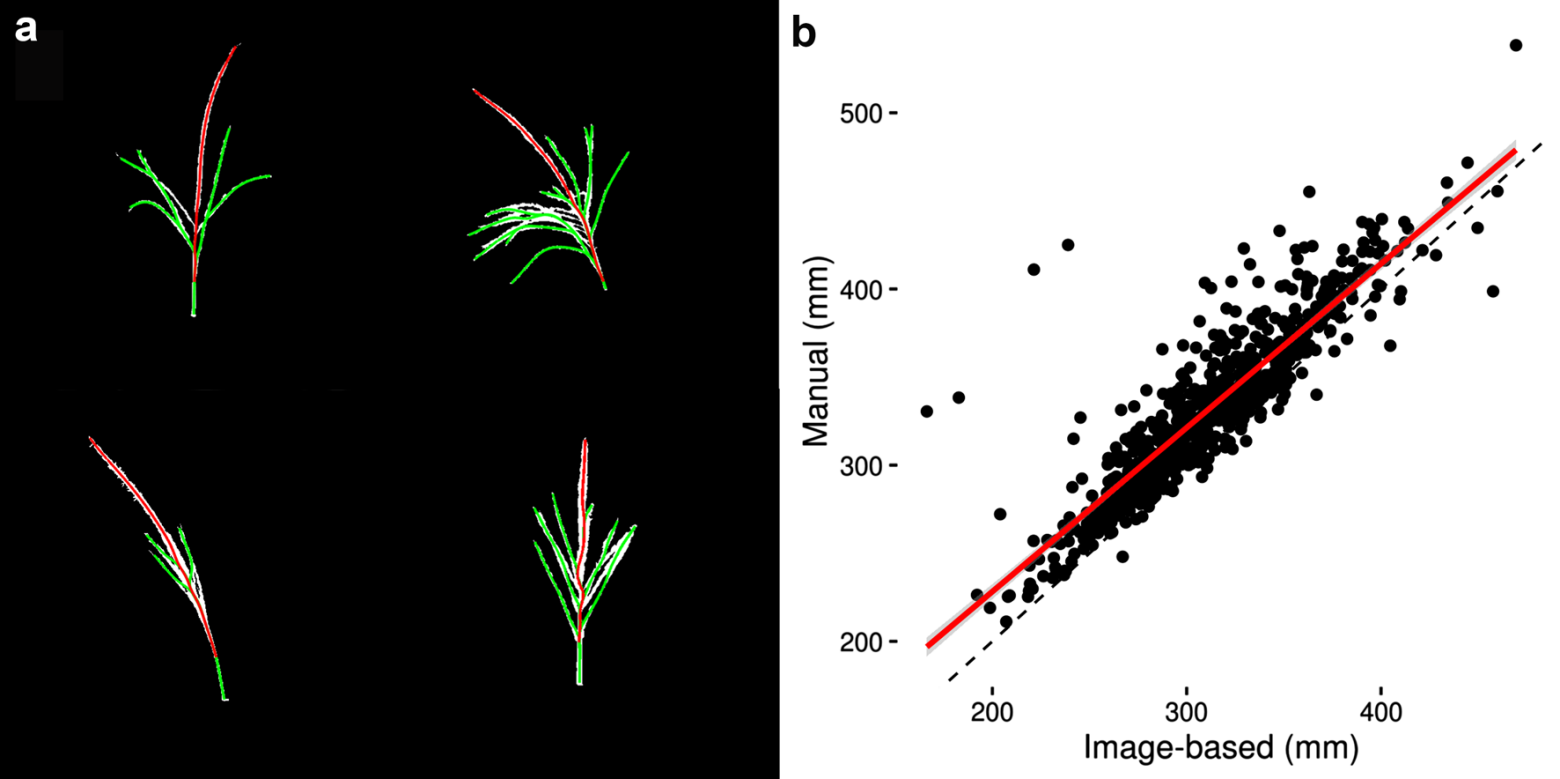
Illustration and scatter plot of tassel length calculation. **a** Example of branch and spike identification for four different tassels. Cubic smoothing splines fit to branches shown in *green*, with the cubic smoothing spline along the main spike shown in *red*. Tassel length is calculated as the line integral of the red spline. **b** Plot of genotype means as obtained by manual and image-based measurement methods. *Red line* represents the fit of the manual onto the image-based measurements, and the *gray shaded region* represents the 95% confidence interval of the fit. The *black dashed line* represents a one-to-one relationship between manual and image-based measurements

Tassel length is typically measured from the lowermost tassel branch to the tip of the central tassel spike. In order to calculate tassel length from the acquired images, the tassel was skeletonized and distances along the skeleton were calculated from the base of the tassel to all other endpoints of the skeleton. The longest identified path was assumed to be that from the base to the tip of the spike. Along this path, the lowest tassel branch was identified, and the distance from that branch point to the tip of the spike was measured, representing tassel length (Fig. 4).

The binary mask of the tassel was first smoothed by convolving it with an isometric two-dimensional Gaussian kernel in order to create a smooth contour and assist the process of isolating a faithful skeleton without spurs. The Gaussian kernel used was 31 pixels by 31 pixels with a standard deviation of 55 pixels, though these parameters are adjustable in the code. The smoothed image was thresholded again to produce a new binary mask with a smooth contour, which was thinned to produce a skeleton. The endpoint of the line network closest to the bottom and center of the image was automatically identified as the base of the tassel, and the graphical distance from the base to each different endpoint was calculated using Dijkstra’s algorithm [16, 17]. Because small protrusions from the tassel can cause the appearance of a short branch, any endpoints closer than 75 pixels to a branch point were excluded from further analysis. The endpoint farthest from the base was assumed to be the tip of the spike. Because tassel length is traditionally measured from the first tassel branch node to the tip of the spike, the lowest tassel branch was identified. Single-row sums of the pixel values in the unsmoothed binary mask were taken within a window of 301 pixels centered on the path from base to spike tip. The sums were smoothed by a Gaussian kernel of width 41 with a standard deviation of 5. The lowest branch was identified as the point along the path where the first derivative of the smoothed sums versus path position was greater than 0.2. Tassel length was calculated as the line integral of a cubic spline fit along the skeleton from the lowest branch to the spike tip. The parameters for the smoothing kernels, minimum skeleton branch length, width of the row-sum window, and threshold for the derivative of the smoothed sums were chosen heuristically and are user-definable.

The correlation between hand and image-based measurements of tassel length was 0.89. This correlation may be driven down by inaccuracy of hand measurements, inability of the image processing algorithms to correctly identify the lowest branch, or by the tilt of the tassels out of the focal plane. Hand-measured tassel length varied more than twofold from 211 to 538 mm, demonstrating large morphological variation which can be quantified by the image-analysis method presented above.

### Branch number

**Fig. 5:**
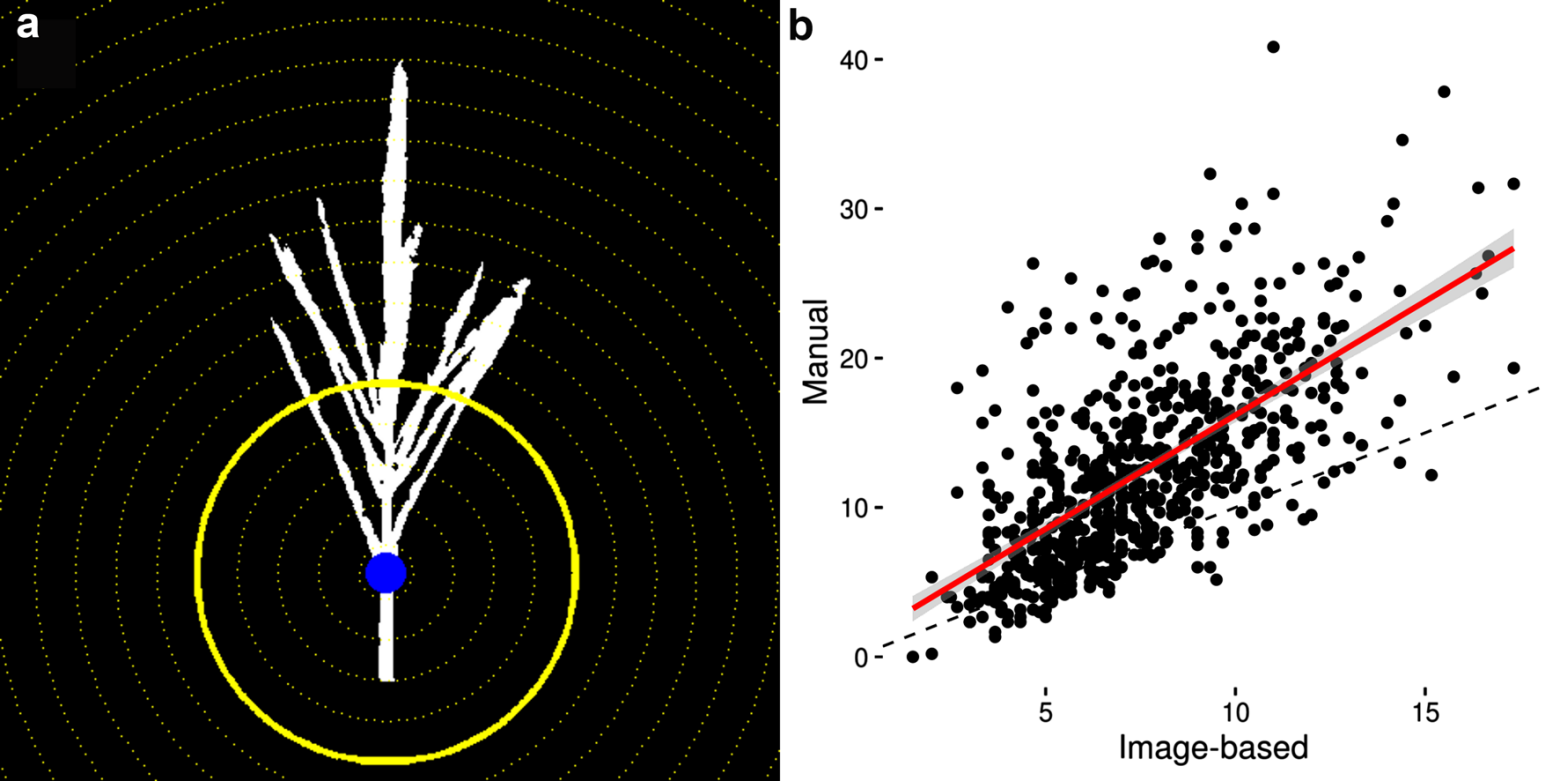
Illustration and scatter plot of branch counting method. **a** Example of branch counting method. Blue circle shows the location of automatically identified lowest branch point. The *solid yellow line* shows the circle with the highest number of intersections with the binary tassel, while *dashed yellow lines* show an example of *circles* with varying radii for which intersections were counted. **b** Plot of genotype means as obtained by manual and image-based measurement methods. *Red line* represents the fit of the manual onto the image-based measurements, and the *gray shaded region* represents the 95% confidence interval of the fit. The *black dashed line* represents a one-to-one relationship between manual and image-based measurements

Another characteristic affecting tassel size is the number of primary branches. Branch number was estimated by centering a circle with a radius of 100 pixels at the lowest branch node, then extending the radius in 50 pixel increments to create a series of circular arcs that intersected the binary object at least once. The number of intersections along each circle was determined and the greatest value was taken as branch number (Fig. 5).

The correlation between hand and image-based measurements of branch number was 0.66, and genotype averages for manually measured branch number ranged from zero to 41. Because manually counting the number of branches on a tassel is likely to be accurate, this low correlation is probably due to overlapped branches being hidden in the image. Such unresolvable occlusions are a limitation inherent to 2D images. A reasonable assessment of the computational method would be to compare branch number values determined automatically from the image by TIPS with the number a human can discern by eye in the same image. This was done with an arbitrary subset of 97 images, each representing a different genotype. The correlation between these two results was 0.83, setting an estimate of the theoretical maximum accuracy for branch number measurement in this context. The automated, image-based circle method in TIPS was unable to count more than 15 branches per tassel in the subset of 97 images, while the maximum number of branches counted by a human in the same subset was 28. These results show that the TIPS has difficulty identifying unique branches when they overlap in the images. Even with the decrease in ability to measure branch number from 2D images, the circle-based automated counting technique implemented by TIPS still yields a correlation of 0.66 with manual measurements, which approaches the theoretical maximum accuracy of 0.83.

### Spike length

**Fig. 6:**
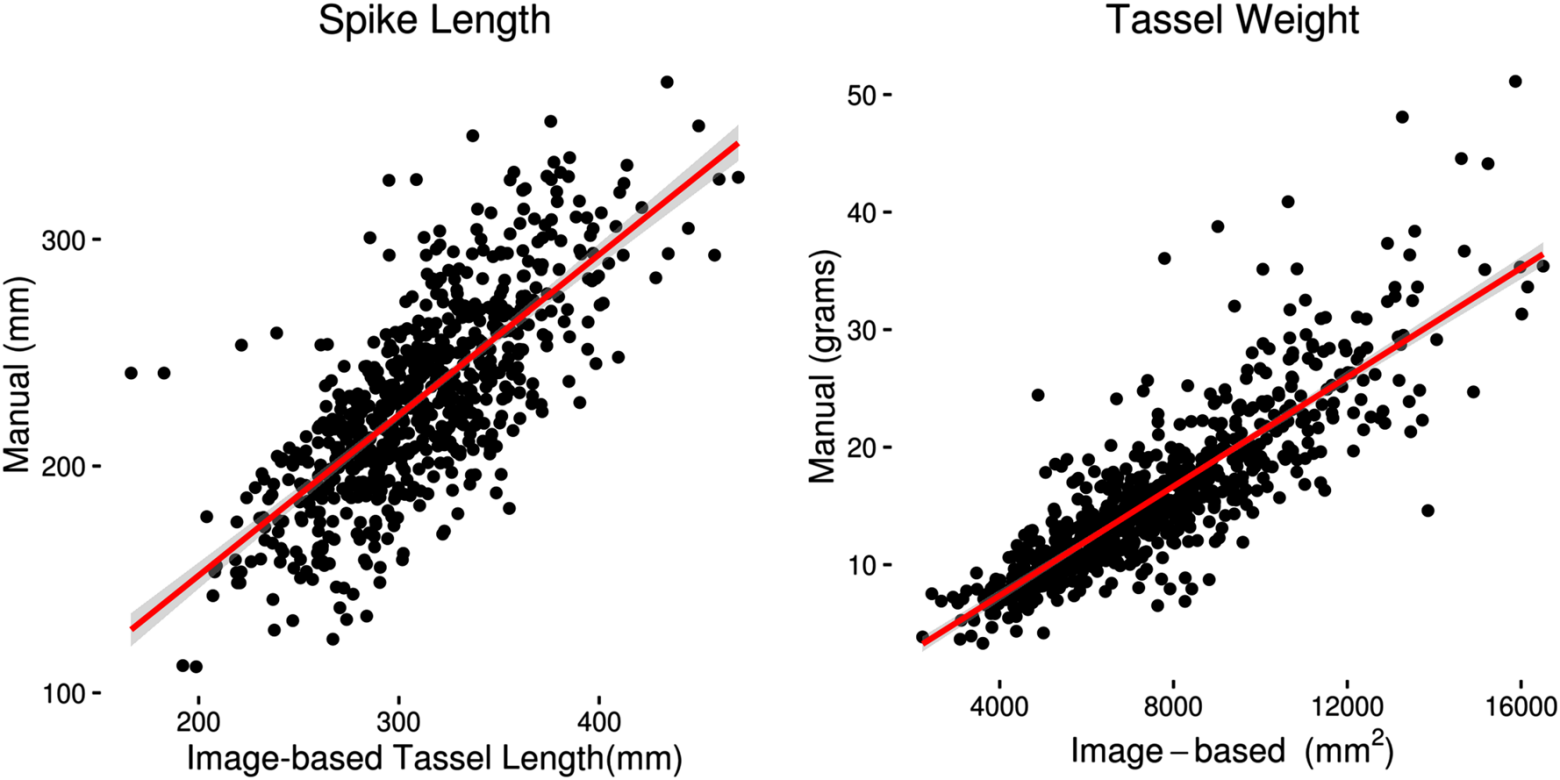
Estimation of spike length and tassel weight from image-based measurements. Plots of genotype means for spike length and tassel weight on image-based tassel length and area, respectively. *Red line* represents the fit of the manual onto the image-based measurements, and the *gray shaded region* represents the 95% confidence interval of the fit

The spike is the segment of the tassel above the topmost branch. In conjunction with tassel length, spike length indicates what proportion of the total tassel is not occupied by branches. A short spike relative to the overall length indicates a correspondingly larger region bearing branches, while a long spike indicates a shorter branching zone. Image analysis methods designed specifically for spike length prove difficult to develop, largely because the highest branch point is more difficult to identify automatically than the lowest branch point used to measure tassel length. Manual measurements of spike length varied threefold across genotypes in the population, ranging from 112 to 369 mm. Although a robust method of directly measuring spike length was not devised, spike length measured manually was found to have a correlation of 0.74 with tassel length measured automatically (Fig. 6). Therefore, automated tassel length measurements can predict spike length reasonably well.

### Tassel size and shape

In addition to the measurements described in preceding sections, the software presented here also returns measurements of tassel area, compactness, tortuosity, fractal dimension, skeleton length, and perimeter.

Tassel dry weight is another measure related to overall tassel size. This is a time consuming measurement to take by hand as it requires drying the samples for a period of time and weighting individual samples. Tassel dry weight was automatically estimated by the image-based method of calculating tassel area as the sum of pixels in the binary tassel mask (Fig. 6). This measure of mass had a correlation of 0.85 with manually measured dry tassel weight.

**Fig. 7:**
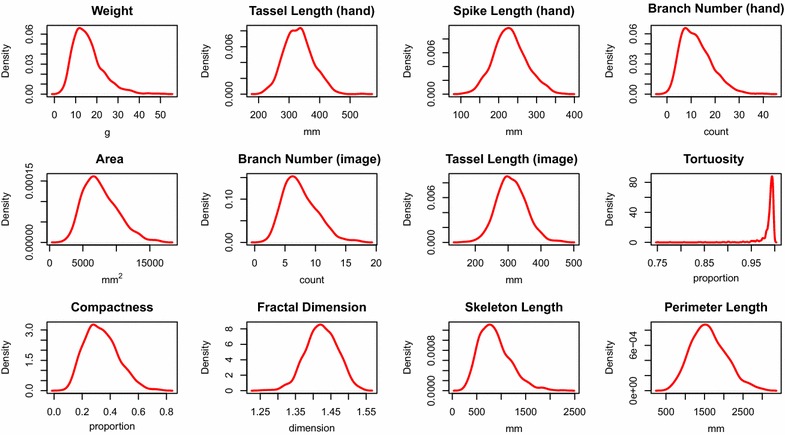
Trait distributions. Density plots of genotype means for all traits described within this paper. *Top row* represents hand-measured traits, while all other plots were obtained from image analysis methods

Compactness was calculated as the tassel area divided by the area contained within a convex hull around the tassel. Compactness represents how densely the tassel’s biomass is arranged. Highly compact tassels would be expected to shade the maize canopy less. Compactness varied greatly across the population (Fig. 7), ranging from 0.07 to 0.76. The ability to quantify compactness precisely may enable tests of the hypothesis that tassel shading affects plant performance by decreasing light interception [18]. The ability to measure compactness automatically could be useful for breeding improved genotypes with reduced tassel shading.

Tortuosity of the main axis was measured as the Euclidean distance between the first tassel branch and the spike tip divided by the tassel length, which produces a measurement between 0 and 1 and gives an indication of the spike’s curvature. Tortuosity is similar to compactness in that it affects the dimensions of the total space occupied by the tassel. Often tassels with curved spikes also have curved branches, and the degree of curvature may be related to structural composition. This type of measurement is difficult to obtain objectively by hand. Tortuosity was the parameter that varied least across the population (Fig. 7).

**Fig. 8:**
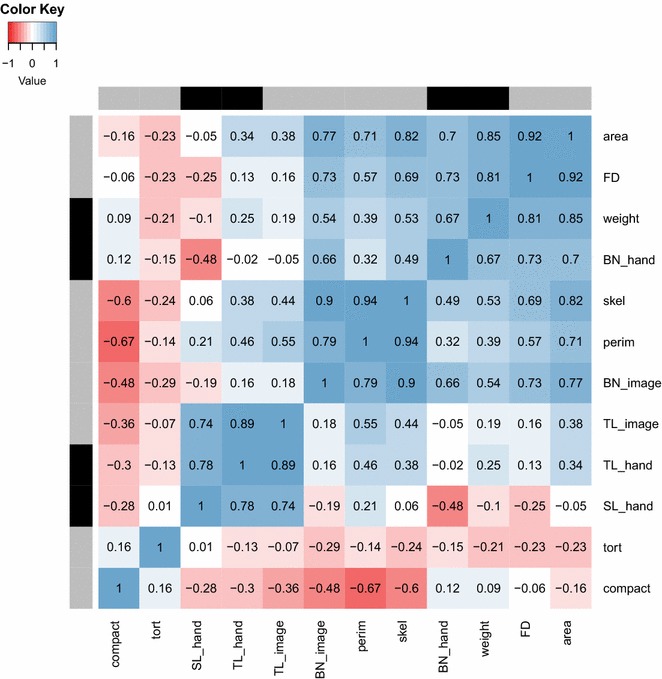
Heatmap of correlations between hand- and image-based measurements. This heatmap shows Pearson correlations between all hand- and image-based measurements described in this study. Traits marked by *black boxes* along the left and top borders were measured by hand, while traits marked by *gray boxes* were extracted from images. FD: fractal dimension; tort: tortuosity; compact: compactness; SL_hand: hand-measured spike length; TL_image: image-based tassel length; TL_hand: hand-measured tassel length; BN_hand: hand-measured branch number; BN_image: image-based branch number; skel: skeleton length; perim: perimeter length

Fractal dimension is a measure of the complexity of the tassel shape. It was estimated using the box-counting method [19]. Its biological significance is not obvious, yet it was highly correlated with tassel weight and branch number (Fig. 8) and varied widely across the population (Table 1).

Skeleton length was measured as the sum of the pixels in the skeleton created during tassel length computations. The perimeter was measured as the sum of the pixels in the outline of the tassel, which was obtained by setting pixels to zero if all their adjacent pixels were equal to one. Both these measurements are indicative of overall tassel size, and were correlated with hand measured weight (Fig. 8).

### TIPS provides an accurate method for image-based phenotyping of tassels

Traits measured by TIPS show moderate to strong correlations with the traits measured by hand, ranging from 0.66 to 0.89 (Table 2). Manually measured traits were tassel length, spike length, branch number, and tassel weight (Fig. 1). Substantial variation was observed for all measured traits in this set of 749 diverse inbreds (Fig. 7). Trait repeatability ranged from 0.85 to 0.92 for hand measured traits; from 0.77 to 0.83 for image-based measurements of area, tassel length, and branch count; and from 0.49 to 0.81 for other image-based measurements for which no hand measurements were available (Table 1). These results suggest that TIPS is capable of accurately measuring morphologically diverse materials.

**Table 2:** Correlations and coefficients of variation of root mean squared error for measured traits

Trait	r	CV(RMSE)
Tassel weight	0.85	0.24
Tassel length	0.89	0.06
Spike length	0.74	0.13
Branch number	0.66	0.38

Correlations (r) and coefficient of variation of root mean squared error [CV(RMSE)] between hand-measured and image-based phenotypic values for four traits in the WiDiv, calculated based on genotype means across two field replications

In some cases, traits that were only computed on images show higher correlations to related hand measured traits than the image-based methods for those traits. For example, fractal dimension has a higher correlation than image-based branch count to hand-measured branch number. However, the reported correlations in Table 2 are between hand-measured traits and the image-based methods that were designed specifically to quantify them. This was done because the image-based methods were designed to have tractable biological meaning with relation to their hand-measured counterparts. High correlations between manual and image-based measurements of tassel length and tassel weight, along with relatively strong correlations with traits that are hard to quantify in two-dimensional images (spike length, branch number), support the claim of TIPS as an accurate method for morphological evaluation of diverse genotypes (Fig. 8). Thus, these results demonstrate TIPS can be used to accurately quantify other traits that are difficult to measure manually. Computer-generated phenotypes and immortal images allow future detailed genetic dissection of tassel morphology at the convenience of the researcher, as opposed to manual measurements which may need to be performed during a narrow time window while the plants are alive.

### Comparison with other image-analysis software

GiA Roots [10] was unable to process all of the images, successfully returning image-based phenotypes for 197 of 200 images submitted. Traits measured by GiA Roots include traits similar to those measured by TIPS, such as maximum and median number of roots (similar to branch number), network area (similar to area), network perimeter (similar to perimeter length), network solidity (similar to compactness), and network length (similar to skeleton length). Of the traits returned by GiA Roots the highest correlation to any of the manually measured traits was 0.40, indicating poor adaptability to tassel images. Though GiA Roots was able to threshold and binarize the tassel images, we hypothesize the width of tassel branches and variability due to spikelets contribute to inaccurately complex skeletonization, which causes imprecise trait measurements.

DIRT [11] computes a large number of traits corresponding to root length and width, lateral branch frequency and length, angles between central and lateral roots, root density, spatial distribution, and biomass accumulation. DIRT thresholded and processed the images appropriately, but produced measurements that had lower correlations to tassel weight, tassel length, and spike length compared to the results of TIPS. DIRT was better able to predict branch number in this subset of images than our methods (Table 3). None of the best-correlated DIRT traits have any clear biological relationship with the tassel traits to which they are correlated.

**Table 3:** Comparison of an existing image-analysis software DIRT to TIPS

Trait	DIRT	TIPS	p value
Tassel weight	0.89 (soil tissue angle 90% 1)	*0.95*	1.0 × 10^−8^*
Tassel length	0.71 (roots seg 2)	*0.91*	1.5 × 10^−12^*
Spike length	0.52 (soil tissue angle 50% 2)	*0.85*	1.3 × 10^−6^*
Branch number	*0.86* (root top angle)	0.81	2.4 × 10^−2^

Correlations between hand-measured and image-based phenotypes for a random subset of 200 tassel images. ‘DIRT’ column represents highest correlation with traits output by DIRT (trait name as output by DIRT in parentheses). ‘TIPS’ column represents the correlation with the TIPS method designed for each trait. The higher correlation is Italicized. The p value column represents the result of a two-sided test for equivalence of dependent correlations [13]. p values that are significant at a Bonferroni-corrected threshold corresponding to α = 0.05 for four tests (1.25 × 10^−2^) are noted with an asterisk

PANorama [8] was unable to consistently construct faithful skeletons from the tassel images. Because background artifacts caused problems with skeleton production, tassel images with the background removed from the image were also used as inputs for PANorama, but skeleton construction still failed. We speculate that because it was created for use with flattened and arranged samples, the images of tassels in their natural orientation were not suitable for PANorama.

## Discussion

Advances in high-precision, high-throughput, image-based phenotyping in combination with currently available genomic tools are expected to increase our understanding of the genetic underpinnings of complex traits and accelerate breeding outcomes. This paper presents a novel system that applies fundamental and robust image analysis methods to provide reliable image-based measurement of maize tassels, for which there are currently no dedicated image analysis tools. TIPS was developed to increase the rate of measurement and information content relative to traditionally hand-measured traits, and to calculate other relevant traits (tortuosity, compactness, fractal dimension, skeleton and perimeter length) that are difficult to reliably measure manually. As such, this system needed to be able to faithfully compute traits that are usually hand measured and have fast enough image acquisition to enable imaging of replicated, field-grown populations with thousands of individuals that reach maturity within a relatively narrow window of time. Manual measurements were the most time-consuming step, and without them the throughput could likely be doubled. Compared to other inflorescence imaging software [8, 9], TIPS has a distinct advantage for experiments that need to image tassels without deforming or preserving them.

Accuracy of image-based tassel length measurements could likely be increased by small changes to the positioning of the tassels. Figure 4b shows that the image-based measurements are slightly biased low. If a tassel is not placed perfectly with curvature perpendicular to the camera, the tassel length will be underestimated. In some cases, as shown in Fig. 4a, the tassel length splines (shown in red) are wavier than they should be due to the skeleton being pulled off center by branches or irregular binary image thickness. This can result in overestimation of the tassel length but has a negligible effect, as evidenced by the fact that estimates of overall length tend to be biased low. Minute differences in the angle of the tassel with respect to the camera add noise to the image-based measurements. Because images were captured from a single angle (for the sake of throughput), accuracy of branch number was reduced by occlusion of some branches by other parts of the tassel. This is also apparent in Fig. 5, which shows that the image-based branch number is also biased low. This could potentially be improved by capturing images from more than one angle.

Spike length is determined by the growth of the inflorescence meristem (IM), which produces a set of lateral branch meristems (BMs) that develop into the tassel branches [20]. Overall tassel length is a result of the number and spacing of BMs plus the length of the IM above the final BM. Variation in the relative lengths of the spike and tassel may be important for studying tassel architecture and pollen production. However, TIPS cannot consistently identify the uppermost branch point, necessary for precise calculation of spike length, so spike length is estimated based on overall tassel length alone. Identification of the spike is complicated by tassel branches occluding the uppermost branch point, and is even difficult for a human to perform accurately given only the available 2D images. We have made a subset of 200 tassel images and their associated background images available at http://phytomorph.wisc.edu/download/TIPS for testing, expansion, and improvement of the methods presented here.

Throughput could be increased even farther by developing methods to image tassels without removing them from the plants, though physical hurdles (e.g., tassel orientation) and technical problems (e.g., background removal) may complicate measurements like tassel length and branch number. However, the ability to image a larger number of tassels may counteract some such issues.

There is no other dedicated tool for image-based phenotyping of maize tassels, and no software that can perform measurements of tassels without imposed positioning on a flat surface. TIPS fills a niche in image-based phenotyping of maize, for which current tools can characterize stalk cross-sections, roots, growth stage, ears, and kernels [9, 11, 21–24]. The ability to quantify tassel shape and size beyond simple hand measurements may be helpful in further unraveling the relationships between tassel and ear morphology and between tassel architecture and pollen production. While simple measurements like tassel length and branch number form an important beginning for descriptions of tassel size and shape, the advent of image analysis methods allows description of more complex or nuanced phenotypes (e.g., compactness, tortuosity) that can be used for germplasm description and characterization, as well as mapping studies. The addition of traits that cannot be measured by hand provides data that can be used along with a training set of ground truth measurements from a representative sample of a population of interest to construct models (e.g., using partial least squares) that may increase the accuracy of tassel length, branch number, or spike length estimates. Once tassels have been imaged, those files can be made available for future studies of novel traits, eliminating the need for repeated field experiments that require large resource commitments.

This study presents results that demonstrate image-based analysis can produce faithful measurements of traits that are normally measured by hand and quantify traits that would otherwise be difficult or impossible to measure. We demonstrate the ability of TIPS to measure tassel morphological traits on 3530 individual tassels from 749 unique inbred lines in the context of a diverse population representing the range of tassel shapes and sizes that would be encountered in a modern breeding or academic research program. As plant genetics experiments continue to increase in size, image-based phenotyping tools such as this have the potential to continue to improve phenotype acquisition with the goals of increased throughput and accuracy. The image acquisition system described here will serve as a basis for expansions on the traits described, and provides proof-of-concept that image-based phenotyping can accelerate measurements of maize tassels.

## Additional file

**Additional file 1.** List of parameters accepted by TIPS, their default values, and descriptions.
