# Supplementary material for: TIPS: a system for automated image-based phenotyping of maize tassels
Source: Plant Methods. 2017 Mar 31;13:21. doi: 10.1186/s13007-017-0172-8 (PMC5374692; doi:10.1186/s13007-017-0172-8)
Supplement: Supplementary file 1 — Additional file 1. List of parameters accepted by TIPS, their default values, and descriptions. [file 13007_2017_172_MOESM1_ESM.docx]

**Table S1**

| **Parameter** | **Default Value** | **Description** |
| --- | --- | --- |
| **gThresh** | 0.08 | TIPS uses the MATLAB's *graythresh* function to binarize the image. If the threshold that *graythresh* uses to binarize is < **gThresh**, the image will throw an error. This is in place to help avoid analyzing images with no tassel in the foreground, bad contrast, or reflection. The default was determined empirically using a set of faulty and acceptable images. |
| **padSize** | 200 | The amount of padding in pixels added around the edges of the original and binarized tassel image to prevent certain operations from falling off the edge of the images. |
| **smoothSigma** | 55 | TIPS uses a Gaussian kernel to smooth the binary tassel image. This is the standard deviation of that kernel in pixels. |
| **smoothKernelDim** | 15 | This is used to determine the size of the Gaussian kernel. The kernel is square with dimension **smoothKernelDim** * 2 + 1 pixels |
| **skelTol** | 10e-8 | Tolerance parameter passed to MATLAB's *csaps* function. |
| **skelMinBranch** | 75 | After the tassel has been skeletonized, any branches with length < **skelMinBranch** pixels will be ignored during spline fitting. |
| **spikeWidth** | 301 | When searching for the lowest branch, TIPS integrates the binary image along the longest spline (corresponding to the tassel spike) within a window of **spikeWidth** pixels centered on the spline. |
| **spikeTol** | 0.2 | The position where the derivative of the integral along the spike spline (see above) becomes > **spikeTol** is identified as the lowest branch point. |
